# Critical Evaluation of the 2016 SOSORT Clinical Guidelines on the Detection and Clinical Management of Adolescent Idiopathic Scoliosis Using the AGREE II Tool: A Chiropractic Perspective

**DOI:** 10.1016/j.jcm.2024.08.001

**Published:** 2024-10-02

**Authors:** Isabelle Pagé, Marie-Joëlle Doré, Chantale Doucet

**Affiliations:** a Department of Chiropractic, Université du Québec à Trois-Rivières, Trois-Rivières, Québec, Canada; b Research Group on Neuromusculoskeletal Disorders, Université du Québec à Trois-Rivières, Trois-Rivières, Québec, Canada; c Center for Interdisciplinary Research in Rehabilitation and Social Integration (Cirris), Centre Intégré Universitaire de Santé et de Services Sociaux de la Capitale-Nationale (CIUSSS-CN), Québec city, Québec, Canada

**Keywords:** Practice Guideline, Scoliosis, Chiropractic, Conservative Treatment, Complementary Therapies

## Abstract

**Objective:**

The objective of this study is to assess the methodological quality of the 2016 Society on Scoliosis Orthopaedic and Rehabilitation Treatment (SOSORT) guidelines for the detection and management of adolescent idiopathic scoliosis.

**Methods:**

A diverse group of experts, including practicing chiropractors, chiropractors with a Diplomate by the American Chiropractic Board of Radiology, chiropractors with PhD degrees, and chiropractors involved in teaching within chiropractic programs was invited to participate in the study. Experts independently evaluated the guidelines using the Appraisal of Guidelines for Research and Evaluation II tool. Individual scaled scores were calculated for each item and the 6 domains, with methodological quality considered good if the score was equal to or greater than 70%.

**Results:**

The 19 experts, including 6 clinicians, 4 chiropractic radiologists, 6 researchers, and 3 lecturers, represented Canada (*n* = 3), United states (*n* = 4), France (*n* = 2), Australia (*n* = 1), United Kingdom (*n* = 1), Switzerland (*n* = 1), Sweden (*n* = 3), Denmark (*n* = 2), China (*n* = 1), and Côte d'Ivoire (*n* = 1). Domain scaled scores ranged from 38% to 90%. The 4 items of the applicability domain scored between 26% and 45%. The experts made suggestions for improving the applicability of the guidelines’ recommendations.

**Conclusion:**

The methodological quality of the 2016 SOSORT clinical practice guidelines was deemed good. However, strategies should be devised to improve their applicability. This could be achieved by involving a more diverse representation of healthcare professions in the development of future guidelines.

## Introduction

Scoliosis represents the most prevalent pediatric spinal deformity, characterized by a 3-dimensional alteration of the spine which affects the coronal, sagittal, and axial planes.^1^^,^^2^ Approximately 80% of cases in individuals aged 10 to 16 years are classified as Adolescent Idiopathic Scoliosis (AIS), with an estimated prevalence of 5% among adolescents.^1^^,^^2^ The precise etiology of AIS is not yet not fully understood, but it is believed to be linked to a combination of factors, including genetic influences as a hereditary trait, systemic metabolic and endocrine disorders, and a possible disturbance in melatonin synthesis.^1^^,^^2^ While only a small percentage of AIS patients presents curvatures exceeding 45° and could, therefore, be candidates for spinal surgery to enhance vital functions, the impact of AIS is still significant. Irrespective of the degree of spinal curvature, individuals with AIS can experience a substantial level of disability, elevated pain levels, and diminished self-esteem due to the aesthetic deformity.^3^

Adolescents and children seeking care for musculoskeletal conditions, such as scoliosis, are likely to receive treatment from a diverse group of healthcare professionals. This group may include family physicians, physiotherapists, chiropractors, and osteopaths.^3^^,^^4^ It is estimated that musculoskeletal conditions account for up to 40% of office visits to healthcare providers.^5^, ^6^, ^7^ Conservative treatment is typically the preferred approach for the majority of patients with AIS. The primary goal of this approach is to halt the progression of spinal curvature, address respiratory problems, alleviate spinal pain, and improve the patient's aesthetic appearance and self-esteem.^3^^,^^8^^,^^9^ Healthcare providers may employ a wide range of assessment and therapeutic methods when managing young patients with spinal pain or deformities. These methods may include, but are not limited to, postural or scoliosis-specific exercises, manual or soft tissue therapy, education, counseling, and the use of braces.^3^^,^^8^, ^9^, ^10^

Clinical practice guidelines are a crucial component in helping healthcare professionals make informed decisions in specific clinical scenarios.^11^ The Society on Scoliosis Orthopaedic and Rehabilitation Treatment (SOSORT), an international scientific expert group, initially introduced guidelines for the conservative management of AIS in 2005^12^ and subsequently updated them in 2011.^13^ Ongoing advances in clinical research made it necessary to revise these guidelines in 2016,^3^ to incorporate the latest findings. The most recent iteration of the SOSORT guidelines features several notable updates, which include demographic insights into AIS, comprehensive descriptions of conservative treatment options tailored to diverse patient populations, thorough literature reviews, and recommendations pertaining to the assessment of adolescents with AIS, prescription of scoliosis-specific orthotics, and physiotherapy exercises, among other conservative therapeutic modalities. In total, this updated guide includes 68 recommendations, organized by relevant domains.

When new or updated guidelines are introduced, several important considerations come to the forefront to ensure methodological quality and successful implementation of the resulting recommendations. For instance, an independent assessment of guidelines methodological quality should be considered, using a validated instrument such as the Appraisal of Guidelines for Research & Evaluation (AGREE) tool.^14^^,^^15^ Moreover, the applicability of the recommendations may benefit from an evaluation of the guidelines by the different stakeholders.^11^

Chiropractic care is frequently sought by the parents or guardians of children, including those with spinal deformities.^4^^,^^7^^,^^16^^,^^17^ In line with this, the 2016 SOSORT guidelines were developed with input from various healthcare professionals, including chiropractors, suggesting that a chiropractic perspective may have been included.^3^ However, detailed information regarding the number of chiropractors involved, their level of expertise, or their specific roles (e.g., clinician vs. researcher) was not provided. Therefore, it seems pertinent to gather insights from an international panel of key stakeholders within the chiropractic profession to assess both the methodological quality of these guidelines and the applicability of their recommendations to chiropractic practice. This would contribute to the literature by highlighting areas that could benefit from further refinement or clarification from a chiropractic perspective. This study aimed to assess the methodological quality of the 2016 SOSORT clinical guidelines using the AGREE II tool and to evaluate the applicability of the resulting recommendations to the chiropractic profession. It was hypothesized that the methodological quality would be considered good overall, but that experts would assign lower scores to the applicability domain compared to other domains.

## Methodology

### Study Design

The study employs a cross-sectional methodological evaluation design where a diverse group of experts assess the quality and applicability of the 2016 SOSORT clinical guidelines.

### Ethics

This study was approved by the Université du Québec à Trois-Rivières Human Research Ethics Committee (CER-19-260-07.17), and all participating experts provided their written informed consent prior to their involvement.

### Experts Recruitment

An international panel of experts was assembled to carry out a rigorous evaluation of the SOSORT clinical guidelines. To ensure a comprehensive representation of the global chiropractic profession, efforts were made to recruit experts from diverse countries. In addition, the recruitment aimed to encompass four distinct categories of experts, reflecting the primary fields in which chiropractors operate worldwide. To achieve these goals, the research team first compiled a list of potential experts from their own networks, selecting individuals who were believed to meet the inclusion criteria. These individuals were then invited to participate in purposive sampling. Subsequently, these experts were asked to recommend additional candidates from their own networks to cover the required categories (snowballs sampling methods). Both IP and CD were actively involved in international groups related to pediatric clinicians and researchers (CD) or chiropractic researchers (IP).

The eligibility and categorization of potential experts were determined through an online questionnaire (SurveyMonkey, San Mateo, CA), a process initiated after securing their informed consent to participate in the study. To qualify, all experts were required to hold a professional chiropractic degree and meet the inclusion criteria corresponding to 1 of the 4 expert categories (refer to Table 1). When an expert could be affiliated with 2 categories, they were instructed to select the category in which they devoted the greatest number of hours per week. Potential experts were excluded from participation if they disclosed any affiliations or relationships with the SOSORT clinical guidelines or the SOSORT group.

**Table 1:** Definition of the Expert Categories and Their Inclusion Criteria

Expert Category	Inclusion Criteria
Clinicians	▪ Chiropractors in practice for at least the past 5 years; work at least 10 hours per week. ▪ Self-identifying with an approach that integrates the best available evidence alongside clinical experience and patient preferences in clinical practice. ▪ Clientele includes children (under 18 years-old).
Researchers	▪ Hold a PhD (or is currently undergoing a PhD degree). ▪ Conduct research in the neuromusculoskeletal field. ▪ Have published at least one paper in the neuromusculoskeletal field in a scientific journal within the past two years.
Chiropractic radiologists	▪ Hold a diplomate from the American Chiropractic Board of Radiology (DACBR) or an equivalent. ▪ Regularly analyze spinal X-rays.
Lecturers	▪ Teach in a chiropractic teaching institution and are involved in theory or hands-on teaching on the neuromusculoskeletal topic (including or not adolescent idiopathic scoliosis).

### Sample Size

The developers of AGREE-II recommend that a guideline should be appraised by a minimum of 2, and preferably 4 evaluators, to improve the reliability of the assessment.^14^ However, in this particular study, a larger sample of participants was chosen to further improve the assessment's reliability, and more specifically, the assessment of the recommendations’ applicability to the chiropractic profession. Considering the 4 expert categories, it was estimated that a total of 16 experts would be adequate. Specifically, the recruitment of 6 clinicians, 4 researchers, 4 professors/lecturers and 2 radiologists were minimally targeted. A greater number of clinicians was sought, considering that this category represents the main users of clinical guidelines. A 50/50 ratio of male and female experts was targeted.

### Expert Characteristics

To safeguard the experts’ anonymity, only general characteristics were collected to provide an overview of the expert sample. These characteristics included gender (male; female; prefer not to respond; other, please specify) and age categories (25-34 years; 35-44 years; 45-54 years; 55-64 years). We also obtained the primary affiliations and job titles of the experts to verify their eligibility and categorization. However, it is important to note that only the country of the affiliation was reported, without divulging specific institutional details.

### Quality and Applicability Appraisal

Each expert was instructed to evaluate the methodological quality of the SOSORT clinical guidelines and the applicability of its recommendations to the chiropractic profession, using the AGREE II instrument. This instrument includes 23 items divided into 6 domains: (1) Scope and Purpose (items 1-3), (2) Stakeholder Involvement (items 4-6), (3) Rigor of Development (items 7-14), (4) Clarity of Presentation (items 15-17), (5) Applicability (items 18-21), and (6) Editorial Independence (items 22-23). The experts were instructed to rate their agreement with each item using a 7-point Likert scale (1-strongly disagree to 7-strongly agree) and to comment on their judgment. In addition to the 23 items, the AGREE II instrument includes 2 final items to rate the overall quality of the guideline (1-lowest possible quality to 7-highest possible quality) and whether the guideline should be recommended for use in practice (yes; yes, with modifications; no). For the current study purposes, experts were instructed to specifically evaluate the domain of applicability to the chiropractic profession. The applicability domain identifies the barriers and facilitators to implementation, strategies to improve the adoption of the guideline, and resource implications of applying the guideline. Experts were given 8 weeks to complete their task and extra time was granted if requested.

### Data Analysis

**Fig 1:**
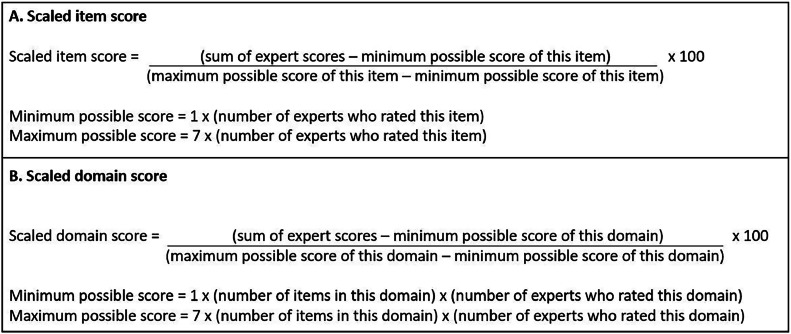
Calculation of the scaled score for (A) the individual items and (B) the domains.

The expert's gender, age and country of affiliations were reported by expert category and for the overall sample. The scaled score of each item and each domain was calculated according to the AGREE II instrument user manual.^14^ To calculate an item scaled score, each expert's rating was summed up for the item considering a value of 1 for “strongly disagree” and of 7 for “strongly agree.” The result was then expressed as a percentage of the maximum possible score for that item, considering the minimum possible score (Fig 1A). A similar procedure was used to calculate each domain scaled score. First, each domain was scored by summing up all the scores of the individual items in the domain. The domain score was then expressed as a percentage of the maximum possible score for that domain taking into account the minimum possible score and the number of experts who had rated that domain (Fig 1B). An item or a domain was considered of good quality if its scaled scored was above or equal to 70%.^14^

### Qualitative Analysis

For each domain, the comments provided by the experts were gathered by 1 of the authors (IP) and divided into 2 categories: those in favor of the guideline meeting the evaluated item and those not in favor. When there was uncertainty about categorization, a second author (CD) was consulted, and a consensus decision was reached. Additional analysis of the comments was performed to emphasize the gaps and shortcomings of items where the scaled score did not meet the threshold for good quality.

## Results

### Expert Characteristics

Of the 32 potential experts who were invited to participate in this study, 7 declined due to lack of time and 2 did not respond to the invitation. From the experts who provided their informed consent to participate (*n* = 23), 79% (19/23) completed the evaluation of the quality and applicability of the SOSORT 2016 clinical guidelines.

Characteristics of the experts per category are presented in Table 2. Overall, 58% (11/19) of the experts were females and most experts (8/19) were aged between 35 and 44 years old. Experts were recruited from Canada (*n* = 3), United states (*n* = 4), France (*n* = 2), Australia (*n* = 1), United Kingdom (*n* = 1), Switzerland (*n* = 1), Sweden (*n* = 3), Denmark (*n* = 2), China (*n* = 1), and Côte d'Ivoire (*n* = 1). The sample included 6 clinicians, 6 researchers, 4 chiropractic radiologists, and 3 lecturers.

**Table 2:** Experts’ Characteristics

	All (*n* = 19)	Clinicians (*n* = 6)	Chiropractic Radiologists (*n* = 4)	Researchers (*n* = 6)	Lecturers (*n* = 3)
Sex					
Women: Men*	11:8	4:2	3:1	3:3	1:2
Age					
25-34 (%, *n*)	26% (5)	17% (1)	50% (2)	17% (1)	33% (1)
35-44 (%, *n*)	42% (8)	17% (1)	50% (2)	50% (3)	67% (2)
45-54 (%, *n*)	26% (5)	50% (3)	0% (0)	33% (2)	0% (0)
55-64 (%, *n*)	5% (1)	17% (1)	0% (0)	0% (0)	0% (0)
Country					
Canada (%, *n*)	16% (3)	17% (1)	25% (1)	0% (0)	33% (1)
United States (%, *n*)	16% (4)	0% (0)	50% (2)	17% (1)	33% (1)
France (%, *n*)	11% (2)	0% (0)	25% (1)	17% (1)	0% (0)
Australia (%, *n*)	5% (1)	0% (0)	0% (0)	17% (1)	0% (0)
United Kingdom (%, *n*)	5% (1)	0% (0)	0% (0)	0% (0)	33% (1)
Switzerland (%, *n*)	5% (1)	17% (1)	0% (0)	0% (0)	0% (0)
Sweden (%, *n*)	16% (3)	17% (1)	0% (0)	33% (2)	0% (0)
Denmark (%, *n*)	11% (2)	17% (1)	0% (0)	17% (1)	0% (0)
China (%, *n*)	5% (1)	17% (1)	0% (0)	0% (0)	0% (0)
Côte d'Ivoire (%, *n*)	5% (1)	17% (1)	0% (0)	0% (0)	0% (0)

⁎ No experts selected the response options “prefer not to respond” or “other.”

### Quality and Applicability Appraisal

Each of the 23 items in the AGREE II instrument was evaluated by all the experts, resulting in complete data without any missing values. Table 3 displays the scaled scores by domain for the entire sample and by expert category, while the scores for each individual item within each domain can be found in the supplemental file. A noteworthy finding was that all but 1 expert recommended the use of the guideline in practice, either as is or with modifications. Pooling together the assessments from all experts revealed that 5 of the 6 domains achieved a scaled score of 70% or higher, indicating good quality. However, the applicability domain only showed a 38% scaled score when assessments from all experts were combined. When results were analyzed within expert categories, there was a variation of up to 30% in scaled scores for the same domain among different expert groups. Overall, when expert categories were not considered, the guideline did not meet the threshold for good quality. Specifically, the researchers' scaled score fell short of the 70% threshold, registering at 58%, while the 3 other expert categories ranged between 71% and 78%.

**Table 3:** Scaled Score by Domain

	All experts (*n* = 19)	Clinicians (*n* = 6)	Researchers (*n* = 6)	Chiropractic Radiologists (*n* = 4)	Lecturers (*n* = 3)
Domains					
1. Scope and purpose	90%	90%	87%	97%	89%
2. Stakeholder involvement	70%	79%	54%	74%	81%
3. Rigor of development	78%	77%	68%	84%	90%
4. Clarity of presentation	78%	79%	74%	79%	85%
5. Applicability	38%	53%	33%	35%	23%
6. Editorial independence	85%	78%	85%	88%	94%
Overall quality	68%	72%	58%	71%	78%
Recommendations					
Yes (%, *n*)	37% (7)	33% (2)	50% (2)	33% (2)	33% (1)
Yes, with modifications (%, *n*)	58% (11)	67% (4)	75% (3)	33% (2)	37% (2)
No (%, *n*)	5% (1)	0% (0)	25% (1)	0% (0)	0% (0)

None of the 4 applicability items reached the threshold of good quality, across all expert categories (Table 4). After pooling the experts’ assessments together, scaled scores varied between 26% and 45%. Clinicians were the ones showing the highest scale scores for the 4 items with 56% for “the guideline describes facilitators and barriers to its application,” 61% for “the guideline provides advice and/or tools on how the recommendations can be put into practice,” 33% for “the potential resource implications of applying the recommendations have been considered,” and 64% for “the guideline presents monitoring and/or auditing criteria.”

**Table 4:** Scaled by Question for Domain 5

Applicability Items	All Experts (*n* = 19)	Clinicians (*n* = 6)	Researchers (*n* = 6)	Chiropractic Radiologists (*n* = 4)	Lecturers (*n* = 3)
18. The guideline describes facilitators and barriers to its application.	40%	56%	28%	54%	17%
19. The guideline provides advice and/or tools on how the recommendations can be put into practice.	42%	61%	42%	33%	14%
20. The potential resource implications of applying the recommendations have been considered.	26%	33%	22%	21%	28%
21. The guideline presents monitoring and/or auditing criteria.	45%	64%	39%	33%	33%

### Comments

Experts provided comments to support their evaluation of most items, with a particular focus on the applicability domain items. Comments related to the applicability domain are presented in the supplemental file. Given that only the items from the applicability domain did not meet the threshold for good quality, we further analyzed only the negative comments related to these items.

Most experts pointed to the absence of discussion about potential barriers to the guidelines' recommendations. For instance, 1 expert commented that “(the guidelines do) not include statements regarding facilitators and barriers to all recommendations contained in the document, such as the ability to provide 1:1 care, the availability of trained scoliosis specialists, or the overall barrier to patients related to the cost of care provided according to the recommendations.” Another expert pointed out that “there is no paragraph on the dissemination/implementation of the guidelines or no additional documents with specific plans or strategies for (the) implementation of the guideline.” With regards to facilitators, experts noted that the guidelines’ publication was open access, that national adaptations were currently under consideration, and that translations into different languages were planned.

Although 1 expert mentioned that “(the guidelines) highlight all the necessary ingredients for the screening, diagnosis and management of scoliosis patients,” experts mainly highlighted the lack of recommendations on the management of spinal pain syndrome and noted that the guidelines were “not very user-friendly.” Experts suggested the need for algorithms, decision trees, flowcharts, quick guides, diagrams, or accompanying materials to support the dissemination and implementation of the guidelines. Some experts made specific recommendations, such as the need for a section that would group “all recommendations together with the specific timing at which they should be implemented,” a “recommendation on the use of X-ray in detail that can be used as a checklist by any chiropractor,” and the need of “lists of sports (recommended or) not recommended due to their impact on spinal mobility.”

Moreover, most experts were unable to find explicit mention of additional resources, such as specialized staff, equipment, or treatment costs, necessary for the application of the guideline recommendations. They mainly pointed to the lack of discussion about the cost of treatment for patients and the cost of equipment for healthcare providers. However, 1 expert commented that addressing resources would have been challenging, considering that the recommendations are intended for global use, and that resource availability varies between countries (e.g., who will be paying for the treatments). Experts also mentioned that, although there was an emphasis on comanagement, “no (information on cost implication (was) easily accessible.”

Comments on monitoring and/or auditing criteria were somewhat contradictory. Some experts noted the guidelines state that “the most important thing is monitoring potential scoliosis progression as well as associated symptoms and quality of life” and that “recommendations include periodic monitoring and auditing markers.” Conversely, other experts highlighted that “there is no paragraph identifying criteria to assess guideline implementation or adherence to recommendations or assessing the impact of implementing the recommendations. In addition, there is no information on advice on the frequency and interval of measurement or descriptions or operational definitions of how the criteria should be measured.” Finally, some experts did identify monitoring and/or auditing criteria, but mentioned that they were “not clearly outlined” or were “very hard to find.”

## Discussion

This critical appraisal study is the first to assess the overall quality and applicability of the SOSORT clinical guidelines for the detection and clinical management of AIS by an independent sample of experts. The findings show that our initial hypotheses were confirmed with regard to the methodological quality of the SOSORT guidelines, as all domains, except the applicability one, reached the threshold for good quality. However, experts representing the chiropractic profession worldwide gave a lower score to the items of the applicability domain.

Clinical practice guidelines (CPG) are considered the gold standard for delivering high-quality healthcare delivery, and clinicians should routinely refer to them when available to inform their clinical decision-making.^18^ However, guidelines’ recommendations have to be based on quality standards that rely on the use of a robust, reliable, and transparent methodology.^18^^,^^19^ As reported in the recent review of Leo et al.^19^ of what is known about CPG development in healthcare, several guides are currently available in the literature to assist CPG development. Nevertheless, once a new CPG is published, external evaluation of its methodological quality should be carried out to ensure that resources for implementation are only assigned to high-quality CPG.^14^ It should be noted that the authors of the 2016 SOSORT guidelines did not mention that their methodology was based on a specific guide, which could have called into question the methodological quality of these guidelines. However, the experts’ appraisal revealed that they considered the methodology used by the authors to develop the recommendations to be appropriate. This can be attributed to the fact that the 2016 version of the SOSORT guidelines was the second update, giving the research team a wealth of experience in guideline development. The ease with which the experts located the information within the guidelines to evaluate the methodology-specific domains highlights the transparency of the SOSORT guidelines development group. Nevertheless, the development group should consider using and reporting on a specific guide if a subsequent update is undergone, which would reinforce the credibility of their methodology. For instance, 5.2% of the experts in the current study (*n* = 1) did not recommend the use of these guidelines, while 57.9% (*n* = 11) believed that modifications were necessary before their implementation. The use of a standardized methodology could potentially have reduced the need for modifications to the guidelines postpublication.

The AGREE II tools define applicability as “the likely barriers and facilitators to implementation, strategies to improve uptake, and resource implications of applying the guideline.”^14^ Based on the low to intermediate ratings provided by the experts for the items in the applicability domain, it appears that the latest SOSORT guidelines do not sufficiently address the applicability of their recommendations. The experts' comments provide a more in-depth understanding of why they found the applicability of the SOSORT guidelines to be limited. In particular, experts highlighted the absence of a clear discussion about potential barriers and facilitators to the implementation of the guideline's recommendations. They also frequently pointed out the lack of a clear discussion about the potential costs and required resources for both patients and healthcare providers. Interestingly, opinions varied regarding the monitoring of patients with AIS, suggesting that information related to this aspect could have been better presented in the guidelines. Furthermore, several experts suggested that additional documents should accompany the main guidelines to facilitate their implementation. Specifically, the experts recommended the development of algorithms, decision trees, flowcharts, quick guides, and diagrams. Extensive evidence demonstrates that healthcare professionals and patients are more likely to benefit from guidelines that include clear implementation tools, as suggested by the experts in this study.^20^, ^21^, ^22^, ^23^ It is worth noting that the earlier version of the SOSORT guidelines included strategies to improve the uptake of recommendations, such as summary tables and flowcharts, which are not present in the 2016 update. To our knowledge, the 2016 SOSORT guidelines are the only available comprehensive resource for the evaluation and conservative management of AIS, making them a key reference for healthcare professionals consulted by parents or guardians of children with spinal deformities. Therefore, the development group should consider creating supportive tools not only to facilitate the implementation of these recommendations by healthcare providers, but also by researchers. A narrative review by Morningstar et al.^24^ highlighted a consistent lack of reported outcomes aligned with SOSORT recommendations in chiropractic studies published between 2000 and 2016.

The results of the current study offer a practical assessment of the methodological quality and applicability of the established gold standard for evaluating and conservatively managing adolescents with AIS. While the expert panel aimed to represent the chiropractic profession, it should be noted that the assessment of the methodological quality would likely yield consistent results, even with a different set of experts evaluating the guidelines. Interestingly, the comments provided by the experts regarding the applicability items were often not specific to the chiropractic profession, making these findings relevant to various healthcare disciplines involved in the conservative care of adolescents with AIS. Future guidelines pertaining to the conservative management and assessment of this spinal deformity should, therefore, consider the findings of this study to enhance the applicability of their recommendations. Furthermore, the identified shortcomings in the guideline's applicability highlight a gap between the recommendations and their practical implementation. Once this disparity is recognized, it becomes both feasible and crucial to develop strategies that bridge this divide. In the near future, the creation of supplementary tools to the SOSORT guidelines to assist clinicians and patients in their decision-making processes would be highly beneficial.

### Strengths

One of the key strengths of this study lies in the global distribution of the experts. The diversity of their practice locations across different continents, each with its unique healthcare context, contributes to a more comprehensive representation of the chiropractic profession.

### Limitations

A limitation of this study is the small number of respondents in each expert category. The inclusion of a larger number of respondents in each category would have been beneficial to enhance the robustness of the results and potentially identify variations in the assessment of applicability items based on the experts' geographical contexts. Consequently, it is important to note that specific comments or ratings provided by the experts may be influenced by country-specific factors, such as treatment costs or clinician specialization. Additionally, the researchers' generally lower ratings for certain domains may be attributed to their advanced skills in searching and critically appraising scientific literature. This divergence in the assessment of the 2016 SOSORT clinical guidelines between expert categories warrants further investigation.

### Future Studies

In future studies involving different groups of experts with varying levels of expertise in the critical assessment of clinical guidelines or scientific literature, it would be important to include a standardized preliminary training program.

Future guidelines should include more in-depth discussions on critical resources, treatment costs, patient monitoring, and auditing. To improve their applicability, consideration should be given to creating supplementary tools such as flowcharts, checklists, and diagrams to support clinicians and patients managing AIS. Enhancing collaboration, such as involving stakeholders from various healthcare professions at each phase of guideline development, would be beneficial for future guidelines, including those by SOSORT.

## Conclusion

The evaluation of the 2016 SOSORT clinical practice guidelines by an international chiropractic expert panel confirmed their good methodological quality. However, most experts identified the need to improve their applicability and implementation.

## AI Statement

During the preparation of this work the author(s) used the language model ChatGPT (version 3.5) developed by OpenAI in order to improve readability and language. After using this tool, the authors reviewed and edited the content as needed and take full responsibility for the content of the publication.

## Appendix Supplementary materials

Image, application 1
