# Supplementary material for: Critical Evaluation of the 2016 SOSORT Clinical Guidelines on the Detection and Clinical Management of Adolescent Idiopathic Scoliosis Using the AGREE II Tool: A Chiropractic Perspective
Source: J Chiropr Med. 2024 Oct 2;23(4):162–70. doi: 10.1016/j.jcm.2024.08.001 (PMC11701840; doi:10.1016/j.jcm.2024.08.001)
Supplement: Supplementary file 1 [file mmc1.docx]

Supplementary Table 1. AGREE II tool score by item and scale domain scores.

| **Domains** | **Items** | **AGREE II tool score** | | | | |
| --- | --- | --- | --- | --- | --- | --- |
|  |  | **All experts (n=19)** | **Clinicians (n=6)** | **Researchers (n=6)** | **Chiropractic radiologists (n=4)** | **Lecturers/non-researcher professors (n=3)** |
| Domain 1: Scope and purpose | 1. The overall objective(s) of the guideline is (are) specifically described. | 93% | 97% | 86% | 100% | 89% |
|  | 2. The health question(s) covered by the guideline is (are) specifically described. | 92% | 94% | 86% | 96% | 94% |
|  | 3. The population (patients, public, etc.) to whom the guideline is meant to apply is specifically described. | 86% | 78% | 89% | 96% | 83% |
|  | **Scale domain score** | **90%** | **90%** | **87%** | **97%** | **89%** |
| Domain 2: Stakeholder involvement | 4. The guideline development group includes individuals from all relevant professional groups. | 68% | 69% | 53% | 79% | 83% |
|  | 5. The views and preferences of the target population (patients, public, etc.) have been sought. | 62% | 72% | 44% | 71% | 67% |
|  | 6. The target users of the guideline are clearly defined. | 80% | 94% | 64% | 71% | 94% |
|  | **Scale domain score** | **70%** | **79%** | **54%** | **74%** | **81%** |
| Domain 3: Rigor of development | 7. Systematic methods were used to search for evidence. | 82% | 81% | 67% | 100% | 89% |
|  | 8. The criteria for selecting the evidence are clearly described. | 85% | 89% | 69% | 200% | 89% |
|  | 9. The strengths and limitations of the body of evidence are clearly described. | 65% | 64% | 47% | 79% | 83% |
|  | 10. The methods for formulating the recommendations are clearly described. | 89% | 92% | 81% | 92% | 94% |
|  | 11. The health benefits, side effects, and risks have been considered in formulating the recommendations. | 67% | 72% | 42% | 75% | 94% |
|  | 12. There is an explicit link between the recommendations and the supporting evidence. | 87% | 83% | 94% | 79% | 89% |
|  | 13. The guideline has been externally reviewed by experts prior to its publication. | 59% | 50% | 53% | 67% | 78% |
|  | 14. A procedure for updating the guideline is provided. | 88% | 83% | 89% | 83% | 100% |
|  | **Scale domain score** | **78%** | **77%** | **68%** | **84%** | **90%** |
| Domain 4: Clarity and presentation | 15. The recommendations are specific and unambiguous. | 85% | 92% | 81% | 75% | 94% |
|  | 16. The different options for management of the condition or health issue are clearly presented. | 82% | 72% | 83% | 88% | 89% |
|  | 17. Key recommendations are easily identifiable. | 68% | 72% | 58% | 75% | 72% |
|  | **Scale domain score** | **78%** | **79%** | **74%** | **79%** | **85%** |
| Domain 5: Applicability | 18. The guideline describes facilitators and barriers to its application. | 40% | 56% | 28% | 54% | 17% |
|  | 19. The guideline provides advice and/or tools on how the recommendations can be put into practice. | 42% | 61% | 42% | 33% | 14% |
|  | 20. The potential resource implications of applying the recommendations have been considered. | 26% | 33% | 22% | 21% | 28% |
|  | 21. The guideline presents monitoring and/or auditing criteria. | 45% | 64% | 39% | 33% | 33% |
|  | **Scale domain score** | **38%** | **53%** | **33%** | **35%** | **23%** |
| Domain 6: Editorial independence | 22. The views of the funding body have not influenced the content of the guideline. | 92% | 89% | 89% | 100% | 94% |
|  | 23. Competing interests of guideline development group members have been recorded and addressed. | 77% | 67% | 81% | 75% | 78% |
|  | **Scale domain score** | **85%** | **78%** | **85%** | **88%** | **94%** |
| Overall quality of the guideline | | 68% | 72% | 58% | 71% | 78% |

Supplementary Table 2. Comments provided by the clinicians (C), the researchers (R), chiropractic radiologists (D), and the lecturers/non-researcher professors (L) in regards of the applicability domain items.

| **Item 18 - The guideline describes facilitators and barriers to its application.** | |
| --- | --- |
| In favor | (C1) There were several tables with recommendations.  (C10) This has briefly been addressed (publication in open access, national adaptations will be considered, translation to different languages planned), but no barriers mentioned. (R3*) (page 3) published in the Open Access Journal. The Consensus process, involving professionals from all over the world, (...)In the meantime, single national adaptations should eventually be considered. Translations in different languages have been planned.  (R6) The authors highlight the requirements needed to address the requirements for screening diagnosis and management of scoliosis.  (R9) In the section “Applicability” it is stated that the guidelines are published as Open Access for visibility purposes and that a wide variety of organizations/third party payors may review to gain insights. It is also recommended that the guidelines serve as basis for national documents. In the section about brace treatment, there is a subsection on ‘Team role in bracing’. This subsection discusses recommended behavior in recommendation 3-5 highlighting the team as an essential part of treatment. (D2) The facilitator’s prerequisite/experience/qualification information seems adequately laid out in the recommendation. Practitioner’s qualification for delivering treatment (i.e. brace) is well organized and stated clearly.  (D3) The document does include statements regarding barriers in patient compliance and addresses possible solutions within the recommendations. |
| Not in favor | (C1) A guideline summary would be helpful. (C1) I didn’t see anything that addressed barriers or facilitators for guideline recommendations. (C3) School screenings are generic and not necessarily done by qualified caregivers. Manual therapists experienced in pediatrics and scoliosis should be involved in the chain of care. Pain syndromes due to spinal dysfunction are common and should be managed with manual therapy and besides appropriate scoliosis specific exercises, spinal stabilization exercises and appropriate ergonomics specific to the individual can be utilized to improve quality of life and compliance. (C11) The tittle is not clear enough, otherwise well written, concise and clear, easy to find. Not enough strategies to overcoming barriers. (R1) I was unable to identify any discussion of facilitators and barriers. Within the recommendations, they speak of clinician specialized in conservative treatment of spinal deformities and with the “Team role in bracing” the ideals for this role, but did not expand on how these were sought and what to do about them. I also am concerned with the discussion of bracing without known availability to everyone, including cost inhibitions.  (R6) The concept of facilitators or barriers to application is not stated explicitly though. (R9) The section has no discussion about facilitators and barriers to its application. (R2) There is no paragraph on the dissemination/implementation of the guideline or additional documents with specific plans or strategies for implementation of the guideline.  (R5) Team role in ? (p.17) only refers to MD, CPO and PT. No allied professions are mentioned. (D2) Some specialist qualification is vaguely stated. Ex. “scoliosis expert physician” and “clinicians specialized in conservative treatment of spinal deformity”. Lacks discussion under the heading, “other conservative treatment”.  (D3) The guideline includes a statement regarding accessibility of the document, however does not include statements regarding facilitators and barriers to all recommendations contained within the document, for instance the ability to provide 1 :1 care, the availability of trained scoliosis specialists, or the overall barrier to patients related to the cost of care provided per the recommendations.  (D1) Depends on the recommendation. Strategies to overcome barriers are generic and not very specific, i.e. “can be achieved through education” not very helpful!  (D4) I was not able to find that information, however, there is a sentence in the Applicability section saying that this guideline will be visible and accessible to the community.  (L1*) A few comments sometimes about the fact that MDs are not trained as required by the recommendations if not more or less info. (L2) I could not find information relating to the identified facilitators or barriers to the implementation of this guideline. The guideline states that it should be used as a basis for national guidelines and their development. No methods are described in obtaining details of facilitators and barriers. No information is presented with regards to the types of facilitators/barriers. No information is provided on how facilitators/barriers have influenced the guideline. For this section, none of the criteria are fulfilled.  (L3) Seems weak at best. Some statement of publication in an open access journal, Scoliosis and Spinal Disorders, but difficult to find more clarity to facilitators and barriers. |
| **Item 19 - The guideline provides advice and/or tools on how the recommendations can be put into practice.** | |
| In favor | (C3) The guidelines address screening, exercise, quality of life and bracing recommendations.  (R6) The authors highlight all the necessary ingredients for the screening diagnosis and management of scoliosis patients. However, these are embedded within a lengthy guideline document.  (R9) In the section “Applicability” it is stated that the guidelines are published as Open Access for visibility purposes and that a wide variety of organizations/third party payors may review to gain insights. It is also recommended that the guidelines serve as basis for national documents. The section on “Brace treatment” lists 15 recommendations on the management of idiopathic scoliosis with corrective braces. |
| Not in favor | (C3) The guidelines [do not] address [ ] recommendations on the management of spinal pain syndromes. Clinically this has important implications for the child as well. (C10) This is lacking. It is supposed to be targeted to all professionals and their patients, but it is not very user friendly. Flowcharts, quick guide or something like that could be handy.  (C11) A diagram of “if [the spinal curve degree is] above then [this should be done], if below then [this should be done]” could help summarize. (R1) Aside from being published in an open access journal, I did not find that this guideline gave other recommendations. It was a thorough guideline jammed packed with good information; however, it would also need advice and tools on the best way to put into practice for clinicians to easily use. (R2) Other than a paragraph on applicability that describes [open-access] publication and passive dissemination, there is no paragraph on implementation of the guideline nor any specific accompanying materials that have been produced to support the dissemination and implementation of the guideline. (R3*) No specific advice, no algorithm, no additional documents. (R5) Yes, but again allied professions are excluded.  (R6) The inclusion of algorithms, decision trees/rules etc. would allow the recommendations to put into practice more easily.  (R9) However, there is no discussion on this can be put into practice. Otherwise the guideline does not contain any description on how the recommendations can be put into practice. (D3) The guideline provides advice on treatment methodologies and the various providers who should be implementing treatment protocols but does not provide tools, links, or specific summary documents. Though it may be unreasonable to expect tools for all recommendations, links to trained specialists, lists of sports not recommended due to a definition as a sport which ‘greatly mobilizes the spine,’ and lists of sports which could be recommended may be of benefit to a practicing clinician.  (D4) I guess that the tables are providing recommendations and from my understanding, it’s up to the clinician to know how to understand the various level of evidence and put together the clinical presentation, imaging section and decide which type of treatment he will do. But I was not able to find a specific area in the article providing advice on how those recommendations can be put into practice.  (D1) Journal article is not a finished guideline. This is just a journal article, not a full guideline package. Plus, it is stated that this product should be adapted culturally and translated. I don’t know what score to give – I could choose 4, meaning neutral or not applicable. But, it does not mention anything if you are not an MD, orthotist or physiotherapist, so in this sense, I should give this item a rating of 1. So, in order not to screw up your date, I’ll chose 2. (D2) In “Recommendations on bracing”, recommendation on utilization of X-ray is nowhere being in detail that can be served as a checklist that any chiropractor can follow. Needs to provide a specific Cobb’s angle and a consensus of what’s considered a problematic sagittal angle that needs to be assessed.  (L1*) Some clues in the recommendations themselves. Otherwise, little guidance on implementation (should have a section dedicated to management that groups all recommendations together with the specific point in time when they should be implemented.  (L2) I could not find information relating to how the guideline can be put into practice. No implementation section was provided in the guideline. No additional tools and resources were provided to facilitation application. No directions were provided as to how to access resources. None of the criteria for this item were fulfilled.  (L3) Seems weak at best again. Abstract reports main updates for this version, but easy to manage tools aren’t easily identified. Tables provide easiest summary of info. |
| **Item 20 - The potential resource implications of applying the recommendations have been considered.** | |
| In favor | (L3) Some discussion on resource impacts – costs clearly described in at least 1 section with screening. |
| Not in favor | (C1) I didn’t see this but I might have missed it. (C2) Don't recall cost info. (C10) This has not really been addressed, but since this is supposed to be used globally, it would be very hard to do, since the resources in different countries are very different, e.g. who will be paying for the treatments (patient, health insurance, government etc). (C11) Few mentions, not easily found, more implicit than explicit. (R1) Similar to the facilitator and barrier question. I found limited discussion of how to really implement these ideal guidelines. (R2) There is no paragraph on the dissemination/implementation of the guideline nor any additional documents with specific plans or strategies for implementation of the guideline No cost information have been considered. (R5) Allied health professionals are not included.  (R6) The potential resource applications have not been addressed in this guideline document. Specialized training, equipment and expertise are required to diagnose and manage scoliosis patients. These aspects mentioned but not expanded upon in the guideline document.  (R9) The guidelines do not, as far as I can gather, include a discussion about the potential impact of the recommendations on resources.  (D1) This is difficult since chiropractors are not mentioned in the document. The only discussion regarding costs are about screening procedures.  (D2) There is plentiful emphasis that co-management of a scoliosis expert physician with PT and constructing orthotist for brace usage, however, no cost implication information that’s easily accessible. There is no description of how the information gathered was used to inform the guideline development process.  (D3) Resource implications are not discussed in regard to the majority of recommendations. The “Assessment” section considers the financial costs associated various methods however resources are not discussed with any recommendations on treatment methods. A discussion regarding the availability of resources such as trained providers and treatment facilities is not included.  (D4) I was not able to find that information. (L1) No study on the impact on resources since the international guide does not represent any country-specific reality.  (L2) I could not identify resource implications in this guideline. No information was provided regarding costs, methods about determining costs, description of the costs, influence of the costs on the development of the guideline. The guideline did report cost-effectiveness studies and their findings however no specific recommendations were made with regards to the specific recommendations made here.  (L3) Not easily identified. |
| **Item 21 - The guideline presents monitoring and/or auditing criteria.** | |
| In favor | (C10) This is not a black and white thing, so a bit hard to set up definite monitoring criteria. But it is presented that the most important thing is monitoring a potential progression of the scoliosis as well as associated symptoms and quality of life.  (R3*) Item Assessment page 28 with the recommendations table. (R9) The guidelines describe that monitoring of patient outcomes are important (page 10).  (D2) For bracing recommendations, there are reasonable volume of advices to when (at what degrees) to start and when to stop using. (L3) Yes – recommendations include periodic monitoring and auditing markers – authors include strength, evidence level and references. |
| Not in favor | (C1) This was presented in the article but was very hard to find. It would be valuable to guideline recommendation as a summary. (R1) Within each set of recommendations, there were some compliance statement. I do believe some could have had more details on implementation “It is recommended that therapists implement a compliance system for exercise tracking”.  (R2) There is no paragraph identifying criteria to assess guideline implementation or adherence to recommendations or assessing impact of implementing the recommendations. No information on advice on the frequency and interval of measurement or descriptions or operational definitions of how the criteria should be measured.  (R5) However, allied health professionals (PHP) are not included in the "team".  (R6) Monitoring and auditing criteria are not clearly outlined in the guidelines document. (R9) Otherwise, they do not, as far as I can gather, include anything about monitoring and/or auditing. (D1) There are no direct guideline compliance goals or verification methods stated.  (D2) The lay out could be re-formatted in a flowchart to gain easier understanding for practitioners.  (D3) A statement regarding the monitoring or auditing of the recommendations within the guideline is not included. (D4) I was not able to find information on monitoring criteria for those guidelines, unless, when the authors are describing the various studies talking about monitoring treatment (brace for example). (L1*) No information after reading.  (L2) This guideline did not provide defined criteria by which to implement the recommendations in practice. No measure of application was proposed hence monitoring/ auditing performance of the recommendations in practice is not currently feasible. None of the criteria of this section are presented in the guideline (criteria, impact, frequency and interval of measurement, measurement operational description). |

* Comments from R3 and L1 experts were translated into English by the research team.
